# THE SALUTOGENIC EFFICACY OF THE VOICE-ACTIVATED INTELLIGENT PERSONAL ASSISTANT INTERVENTION: A CASE STUDY

**DOI:** 10.1093/geroni/igae098.3441

**Published:** 2024-12-31

**Authors:** Terence Lau, Angela Leung

**Affiliations:** The Hong Kong Polytechnic University, Hong Kong, Hong Kong; The Hong Kong Polytechnic University, Hong Kong, Hong Kong

## Abstract

Existing Parkinson’s disease (PD) interventions are primarily focused on physical rehabilitation and pathogenic-based. The lack of positive, health-orientated psychosocial interventions prompted the research team to develop a salutogenic VIPA user protocol and examine its preliminary efficacy through an 8-week pilot RCT. This case report selected the participants with the largest sense of coherence-13 item scale (SOC-13) improvement to explore their user experience through the salutogenic perspective. A 32-minute face-to-face, post-intervention semi-structured interview adopting the interpretive descriptive approach was conducted on 31st Jan, 2024. The interviewee was a 63-year-old male, level 3 Hoehn and Yahr Scale with 2 years of PD history, documenting a 100% compliance rate and a 16-point improvement in SOC-13 across all comprehensibility, manageability, and meaningfulness subdomains. The transcripts were coded deductively through thematic analysis referencing the salutogenesis and psychosocial well-being framework. Written consent was signed before the research, and the participant agreed to be audiotaped during the interview. The encyclopedia-like features within VIPA facilitated participants’ comprehensibility by retrieving desired online information. On manageability and psychological well-being, the participant metaphorically described the VIPA intervention as an electric wheelchair for physically disabled people to that of people with Parkinson’s Disease (PWP). Thus, the intervention could benefit the autonomy and environmental mastery domain within psychological well-being alongside the manageability domain. For meaningfulness, the participant further elaborated the VIPA intervention could minimize the difference between PWP and other citizens. The salutogenic-based VIPA intervention can contribute a salutogenic force, lessening the effect of constantly deteriorating PD progression experienced by PWP.

